# Optimizing CFTR modulator therapy management for cystic fibrosis through the ReX platform

**DOI:** 10.3389/fped.2023.1300968

**Published:** 2023-12-19

**Authors:** Karin Yaacoby-Bianu, Malena Cohen-Cymberknoh, David Shoseyov, Tal Lavi, Ana Ostrovski, Michal Shteinberg, Galit Livnat

**Affiliations:** ^1^ Pediatric Pulmonology Unit and CF Center, Carmel Medical Center, Haifa, Israel; ^2^ B. Rappaport Faculty of Medicine, Technion—Israel Institute of Technology, Haifa, Israel; ^3^ Pediatric Pulmonology Unit and Cystic Fibrosis Center, Hadassah Medical Center and Faculty of Medicine, Hebrew University of Jerusalem, Jerusalem, Israel; ^4^ Pharmacy Services, Carmel Medical Center, Haifa, Israel; ^5^ Pulmonology Institute and CF Center, Carmel Medical Center, Haifa, Israel

**Keywords:** cystic fibrosis, CFTR modulators, elexacaftor/tezacaftor/ivacaftor, medication delivery platforms, adherence to chronic treatment, remote patient monitoring

## Abstract

**Background:**

Cystic fibrosis (CF) is a chronic multi-systemic disease that requires a complex daily treatment regimen. Therefore, there is sub-optimal adherence to CF therapies, and it was shown to impact its clinical and economic burden. Cystic fibrosis transmembrane conductance regulator modulators (CFTRm) are high-cost medications that demonstrated significant benefit in clinical trials. The aim of this study was to evaluate the safety, usability, and efficacy of the ReX platform in medication management of CFTRm for the treatment of people with CF (pwCF).

**Methods:**

ReX is a patient engagement platform consisting of a cloud-based management system and a cell-enabled handheld device intended to dispense oral medication into the patient's mouth, following a pre-programmed treatment protocol. It provides real-time adherence data to caregivers and timely, personalized reminders to patients. This is a prospective multi-center open study for pwCFs older than 12 years, who had been prescribed CFTRm [elexacaftor/tezacaftor/ivacaftor (ETI) or tezacaftor/ivacaftor (TI)], and provided consent to use ReX platform to receive CFTRm and record their health condition. Study duration was 12–24 months, with clinic visits where physical examination, body mass index (BMI), and pulmonary function tests were performed, and user experience questionnaires were filled in.

**Results:**

Ten pwCFs from two CF centers in Israel were included. The mean age was 31.5 years (range 15–74 years); eight were taking ETI and two TI. Median adherence to CFTRm was 97.5% (range 70%–100%) in the first year and 94% (range 84%–99%) in the second year, which is higher than the previously reported CFTRm adherence of ∼80%. No adverse events related to the use of the platform were reported. Patients reported ReX to be valuable to their treatment management and user friendly. Estimated mean forced expiratory volume in 1 s (FEV_1_%) increased from 74.4% to 80.8% (*p* = 0.004) over 2 years. Similarly, estimated BMI percentile increased from 53.5 to 59.0 (*p* < 0.001).

**Conclusions:**

Using the ReX platform in medication management of pwCF treated by CFTRm is safe, easy to use, and effective in improving the adherence to treatment and the clinical outcomes. Consequently, this device may potentially reduce costs to healthcare providers. Further larger and long-term studies are required to examine the clinical benefits of the ReX platform.

## Introduction

Cystic fibrosis (CF) is a chronic genetic multi-systemic disease, which requires daily time-consuming treatment regimen that includes airway clearance, oral and inhaled medication, nutritional support, and exercise. The complexity of care has increased for people with CF (pwCF) in parallel with increased life expectancy (1), leading to substantial burden of care and disease monitoring (2). Therefore, there is low to sub-optimal adherence to CF therapies, which was shown to impact the clinical and economic burden of the disease (3, 4). In the last decade, cystic fibrosis transmembrane conductance regulator (CFTR) modulator (CFTRm) therapies targeting the basic molecular defect in CF have been developed and have demonstrated significant improved health outcomes, including better respiratory function, nutritional status, and enhanced quality of life (5). Currently, CFTRm are high-cost medications that pose a significant strain on healthcare budgets globally and lead to delays in funding and disparities within the CF community (2, 6). According to a previous report, adherence to CFTRm, evaluated by mean proportion of days covered for three modulators was only around 0.8 (7).

**Figure 1:**
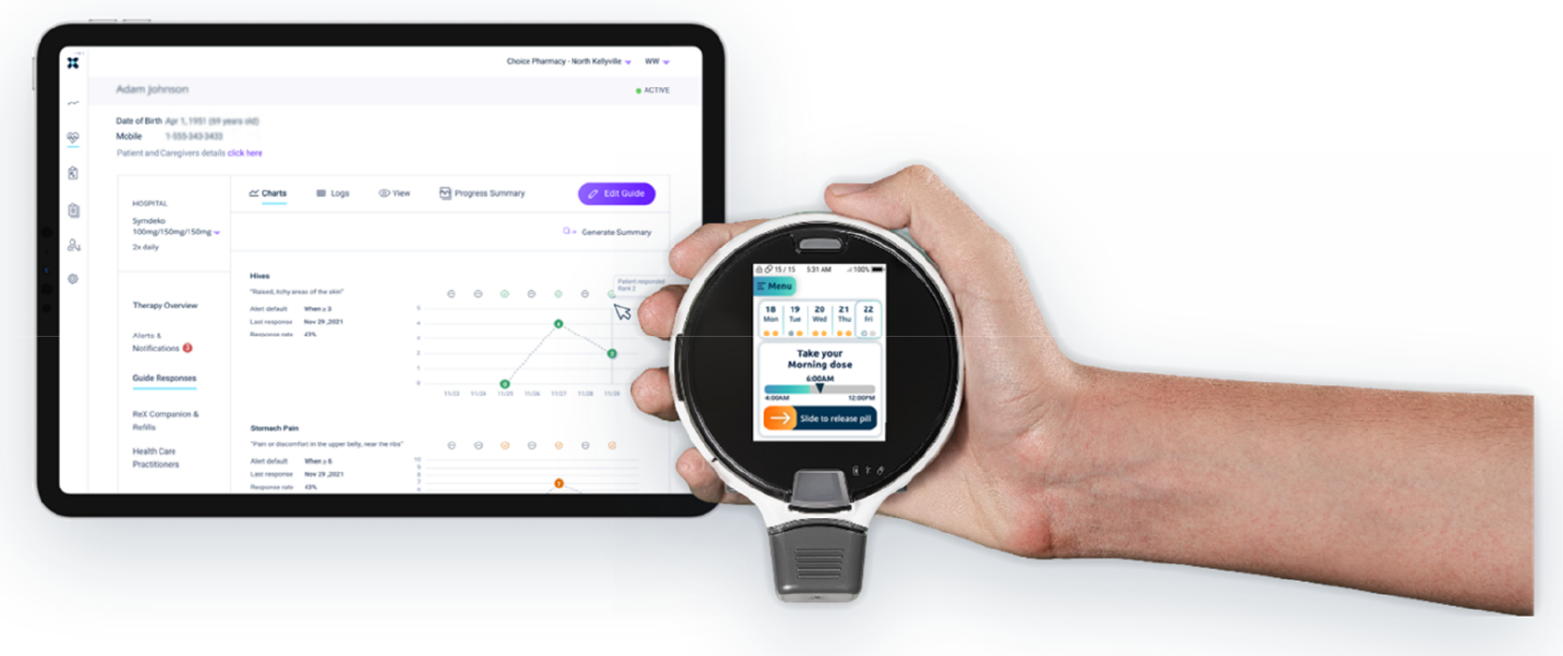
ReX platform.

ReX (Dosentrx Ltd., http://www.dosentrx.com/; Figure 1) is a platform composed of a cloud-based management system and a cell-enabled handheld device. Its primary purpose is to help patients remotely manage their daily medication intake. ReX engages patients utilizing a touchscreen interface and dispenses oral medication into the patient's mouth, following a pre-programmed treatment protocol. In addition, the device is designed to ask patients customized clinical questions to enable remote patient monitoring. ReX addresses sub-optimal adherence to medication therapy by providing real-time, reliable adherence data to caregivers and timely, personalized reminders to patients via text messages and a call center. A patient's treatment plan can be altered in real time based on the answers the patient provides. ReX's novel “tracking to the mouth” technology was found usable and accepted by subjects in a non-clinical feasibility study (8). The same technology was previously developed as a patient-controlled analgesia device and was proved to be a safe and effective means of administering an oral analgesic for hospitalized patients requiring pain therapy after operation (9, 10).

The aim of this study was to evaluate the safety, usability, and efficacy of the ReX platform in the measurement and management of medication adherence in pwCF receiving treatment with CFTRm. In addition, to assess clinical outcomes following treatment with CFTRm using the ReX platform, as compared to retrospective clinical data obtained during standard of care.

## Materials and methods

### Study design and participants

This was a prospective multi-center open study for pwCF, who had been prescribed CFTRm according to their CFTR mutations [elexacaftor/tezacaftor/ivacaftor (ETI) or tezacaftor/ivacaftor (TI)], and accepted to use ReX platform to receive these medications and simultaneously record their health condition. Two CF centers from Israel participated in the study between July 2020 and July 2023: Carmel Medical Center, Haifa (IRB: 023-20-CMC), and Hadassah Medical Center, Jerusalem (IRB: 0712-19-HMO). Inclusion criteria were pwCF ≥ 12 years old, not with end-stage lung disease (forced expiratory volume in 1 s, FEV_1_ ≥ 40%), treated with CFTRm, and able to swallow pills during training (successfully achieved two administrations of “demo” pills of Tic-Tac sweets by ReX).

Following informed consent and eligibility screening, subjects received training on the ReX platform including “demo” pill intake, how to perform cartridge exchange, and were set up in the system. Subjects received the ReX device to use at home and medication stored by pharmacy on the ReX cartridges for use for the following 8–12 weeks until the next visit, with several doses in their original package in case of a malfunction. Subjects were asked to bring their recently used ReX cartridges and the remaining extra pills for counting each following visit. The study duration was 12–24 months for each subject and included seven bi-monthly visits to the clinic in the first year, and four 3-monthly visits in the second year. In each visit, pill accountability was done to measure the subjects’ adherence. In addition, any adverse effects were registered, and physical examination was preformed, following measurements of body mass index (BMI) and pulmonary function test (PFT). User experience questionnaires were filled at 2, 6, 12, and 24 months by the patients, and questionnaires were also filled at the end of the treatment by the medical team. Retrospective clinical history of these subjects was extracted for 1 year prior to recruitment to the study. These data were compared to clinical data obtained during ReX use.

### The ReX platform

The platform comprises of the following elements:

(1) ReX device (ReX), portable medication dispenser with a touchscreen that communicates with the Remote Therapy Manager via the cellular network.
(2) A locked disposable cartridge, supplied pre-loaded with tablets, filled by the pharmacy. The cartridge includes an integral mouthpiece, which releases the pill into the patient's mouth following application of a short inhalation by the patient.
(3) ReX treatment manager, a web-based application used for the collection and management of real-time patients’ adherence and patient-reported outcome (PRO) data.
(4) Reminders dispatched to patients and caregivers according to a predefined logic (email, SMS, or via support team call): take the CFTRm with a fatty meal, including personalized real-time reminders in any case of a “missed dose” event up to 2 h of delay.
(5) PRO questions, tips, reminders, and groups of questions were presented to patients according to defined logic and are collated in the Remote Treatment Manager. This enables providing messages directly to patients. In this study, the subject answered short clinical surveys regarding dyspnea, cough level, fever, weight loss, phlegm (thickness, color, and amount), abdominal pain, and reminders for other treatments: inhalation and chest physiotherapy.
(6) Thresholds in cases that patient reports exceed a certain threshold. Notifications via email and/or text messages are dispatched to the research team in real time according to a defined logic.

The ReX platform is designed to be used in delivering expensive drugs only. The ReX platform is currently under clinical development so pricing and reimbursement are not established yet, but the distribution model is that the pharmaceutical company will pay for its application with the relevant medication to enhance adherence and prevent drug waste.

### Study assessments

(1) Safety: patient's questionnaires were based on safety questions, on issues encountered regarding overdose incidents, pill dropping, pill deformation upon dispensing, or other incidents (see the Supplementary Material). Questions asked on the medical team questionnaires: *Are you aware of a report of a significant event that did not arrive or arrived too late*? *Has a significant event related to medication administration through the device occurred? Have you received a communication from the patient regarding issues with the device? Have you received any comments or requests from the research pharmacist? Have you ever contacted a patient following a device-generated report?*
(2) The device's usability was evaluated using a Likert-like scale in questionnaires, grading the usability between 1 and 5 (1 being user-friendliest and 5 the lowest) regarding screen instructions, inhale release, replacement of cartridge, device charging, and incident reporting with the device (see the Supplementary Material). These questionnaires were handed on the first year of trial only.
(3) General opinion was evaluated using a Likert-like scale in the patient's questionnaires grading their opinion between 1 and 5 (1: don't agree to 5: most agree) on the following questions: I was able to report incidents easily; Got sense of confidence; Reminders helped me take medications on time; My treatment is more efficient with the device vs. without; Want to continue using the device; Will recommend other patients to use it. Similarly, the medical team also graded their perspectives on the following questions: I felt that the system allows me to closely and efficiently monitor the patient's medical condition compared to routine treatment; I am comfortable knowing that if a patient reports a significant event, I will receive a notification in real-time; I have gained benefits from tracking and/or data regarding the patient's adherence level; I feel that my service to the patient is more efficient with the device than without; I would be interested in continuing to use the system; I would recommend other teams to try the system. These questionnaires were handed in at the end of the first and second years.

We evaluated the relative change in the following parameters (1/2 years after commencing CFTRm via ReX platform vs. a year before, mean values for both time periods were analyzed):

(1) Adherence to treatment: subject's adherence was monitored by the ReX platform. Data from each dose event were saved and transmitted to the cloud**.** These data were double checked by the pill accountability during each visit. Data on the adherence to CFTRm in the year prior to the use of the ReX platform were based on patients’ self-reports and multi-disciplinary care team assessment ranging from very low, low, medium to high.
(2) PFTs were performed according to American Thoracic Society/European Respiratory Society standard (11). Percent predicted values of FEV_1_% were measured using the Global Lung Function Initiative (GLI) equations (12).
(3) BMI was calculated for all patients. For those <20 years of age, BMI was presented as percentile for age, and for those ≥20 years, the corresponding BMI percentile was calculated, facilitating comparisons (13).

### Statistical analysis

FEV_1_% and BMI percentiles for each timepoint were presented as medians (Me) with the first (Q1) and the third (Q3) quartile due to small number of observations. However, means with standard deviations (SD) were also presented for informative purposes only. Comparison across different timepoints was performed using paired Wilcoxon signed rank test, with Holm adjustment for multiple comparisons. Data were visualized using boxplots for paired observations (with connected points belonging to the same patient). The mixed-effect model was fitted to the data with the patient as the random effect to account for correlation of several measurements taken for the same patient and timepoint as a fixed effect. In the case when patients switch medication during the study, such information was added to the model to adjust the effect of using the device in the FEV_1_% and BMI percentile. Then estimated means from the model with standard errors were calculated for each timepoint, adjusted for any changes in medications, with *p*-values calculated using Tukey’s honestly significant difference test. Analysis was performed using R 4.3.1 (A Language and Environment for Statistical Computing. R Foundation for Statistical Computing, Vienna, Austria, 2023).

## Results

Ten pwCFs from two CF centers were included in the study: mean age 31.5 years (range 15–74 years), four patients <18 years, all men by chance (most were low to medium adherent), with variant CFTR mutations. The patients’ demographics and characteristics at baseline are presented in Table 1. Six of the patients were pancreatic insufficient. One patient was naive to CFTRm at the study beginning, but the other nine were previously treated by a CFTRm and switched between them as described in Table 1. During the study period, eight were taking mostly ETI, and two were taking mostly TI. Five subjects completed 2 years in the study, four completed 1 year in the study (two due to late recruitment and end of study, one due to irregular visits to clinic, and one due to a prolonged hospitalization), and one patient withdrew after 8.5 months due to personal request unrelated to the study.

**Table 1:** Baseline characteristics of pwCFs treated in the study.

No.	First mutation	Second mutation	Pancreatic status (PI/PS)	Age at study start (years)	CFRD	CFLD	PA	Other infections or issues	ReX (months)	CFTRm Tx during the study period	CFTRm Tx in the prior year
1	DF508	DF508	PI	28	Y	Y	Y	Nocardia; highly unbalanced CFRD; depression	12	TI switched to ETI after 4 months	LI with poor compliance. Switched to TI after 8 months
2	G85E	D1152H	PS	15	N	N	N	One episode of elevated CPK on ETI	24	TI switched to ETI after 4.5 months	TI
3	G85E	D1152H	PS	16	N	N	N	One episode of elevated CPK on TI, and one episode on ETI	24	TI switched to ETI after 4.5 months	TI
4	DF508	DF508	PI	33	Y	N	Y	Unbalanced CFRD; bronchial artery embolization due to severe hemoptysis	23	ETI	ETI
5	D579G	c.2399A>C	PS	16	N	N	N	None	12	ETI	No
6	G85E	G85E	PI	15	IGT	N	N	Intermittent PA	12	ETI	ETI
7	A455E	2326-1 G>C	PI	74	Y	N	Y	Post CVA, hyperlipidemia, hypertension, osteoporosis, glaucoma, retinal detachment, Ischemic heart disease	14	TI switched to ETI after 9 months	TI
8	3849 + 10kbC>T	L138 ins	PS	53	N	N	N	Obesity, hypertension, OSA, hyperlipidemia	24	TI	TI
9	W1282X	DF508	PI	39	N	Y (Post Ltx)	Y	Chronic immune suppressive Tx	8.5	ETI	ETI
10	G542X	DF508	PI	24	N	Y (Post Ltx*2)	N	Chronic immune suppressive Tx	24	ETI	ETI

CFRD, CF related diabetes; CFLD, CF liver disease; F, female; M, male; PA, chronic infection with *Pseudomonas aeruginosa*; PI, pancreatic insufficient; PS, pancreatic sufficient; Tx, treatment; N, no; Y, yes; IGT, impaired glucose tolerance; CPK, creatine phosphokinase; CVA, cerebrovascular accident; Ltx, liver transplant; OSA, obstructive sleep apnea.

*Gender: all 10 subjects were men.

**Figure 2:**
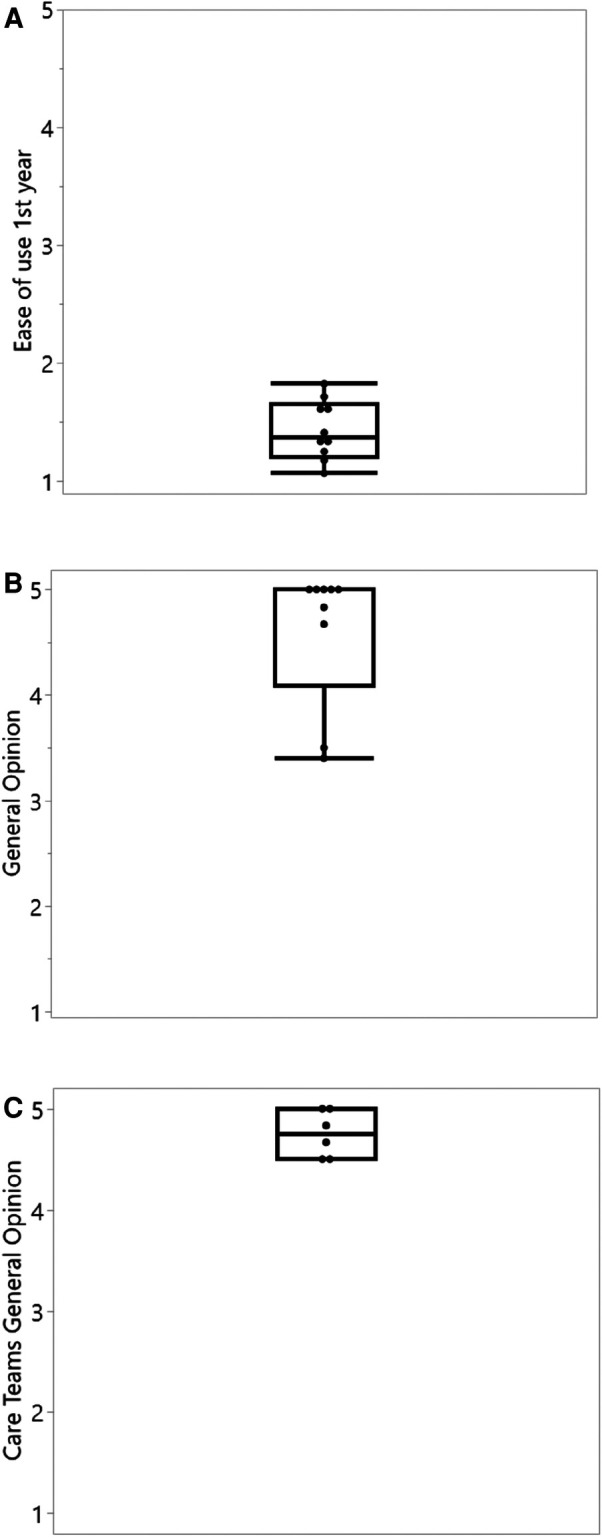
Ease of use and general opinion questionnaire results of the ReX platform. (**A**) Average ease of use questionnaires during the first year of study. 1 = most user friendly (*n* = 10). (**B**) General opinion of patients, last available observation at 12 or 24 months. 5 = most valuable to the treatment management (*n* = 9, one patient withdrew before completing the first year). (**C**) General opinion answered by different care team members upon trial ending. 5 = most valuable to the treatment management (*n* = 6).

Regarding safety, a total of 128 safety questions were answered by the 10 patients, and 36 questions were answered by the two medical teams, showing no adverse events related to the use of the ReX platform. Usability was evaluated by 141 questions to patients at the end of the first year and demonstrated a median of 1.4 (1 being user-friendliest) (Figure 2A). The median general opinion upon conclusion of the trial was scored “5” by patients (Figure 2B) and “4.75” by care teams (Figure 2C).

A total of over 14,500 tablets were taken through the device. This represents a median adherence to CFTR modulators of 97.5% (range 70%–100%) in the first year of study and 94% (range 84%–99%) in second year, which are higher treatment rates compared to pre-ReX clinical assessment of compliance (Table 2) and are higher than the previously reported CFTRm adherence of ∼80% (7).

**Table 2:** Adherence to CFTR modulators.

No.	Year prior to study^a^	Year I (%)	Year II (%)
1	Medium	98.9	—
2	Medium	98.7	94
3	Medium	97	92
4	Medium	97	98
5	Very low	98	—
6	Very low	70	—
7	High	100	—
8	High	99.5	99
9	Medium	97	—
10	Low	93	84
Median		97.5	94

For subject 5 (CFTRm naive), the overall CF treatment adherence was assessed by him and the care team.

^a^ Based on patient self-reports and care team assessments.

**Figure 3:**
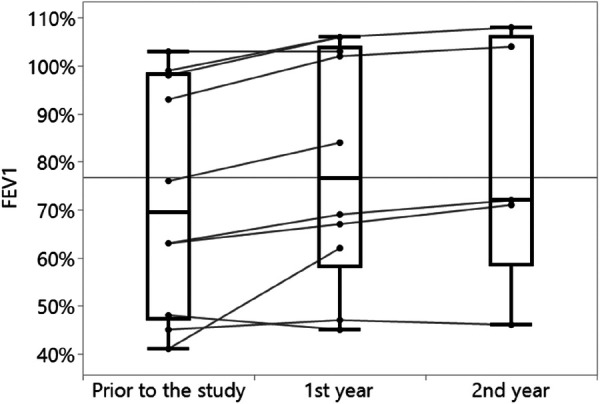
Changes in FEV_1_% over time (*n* = 10).

**Figure 4:**
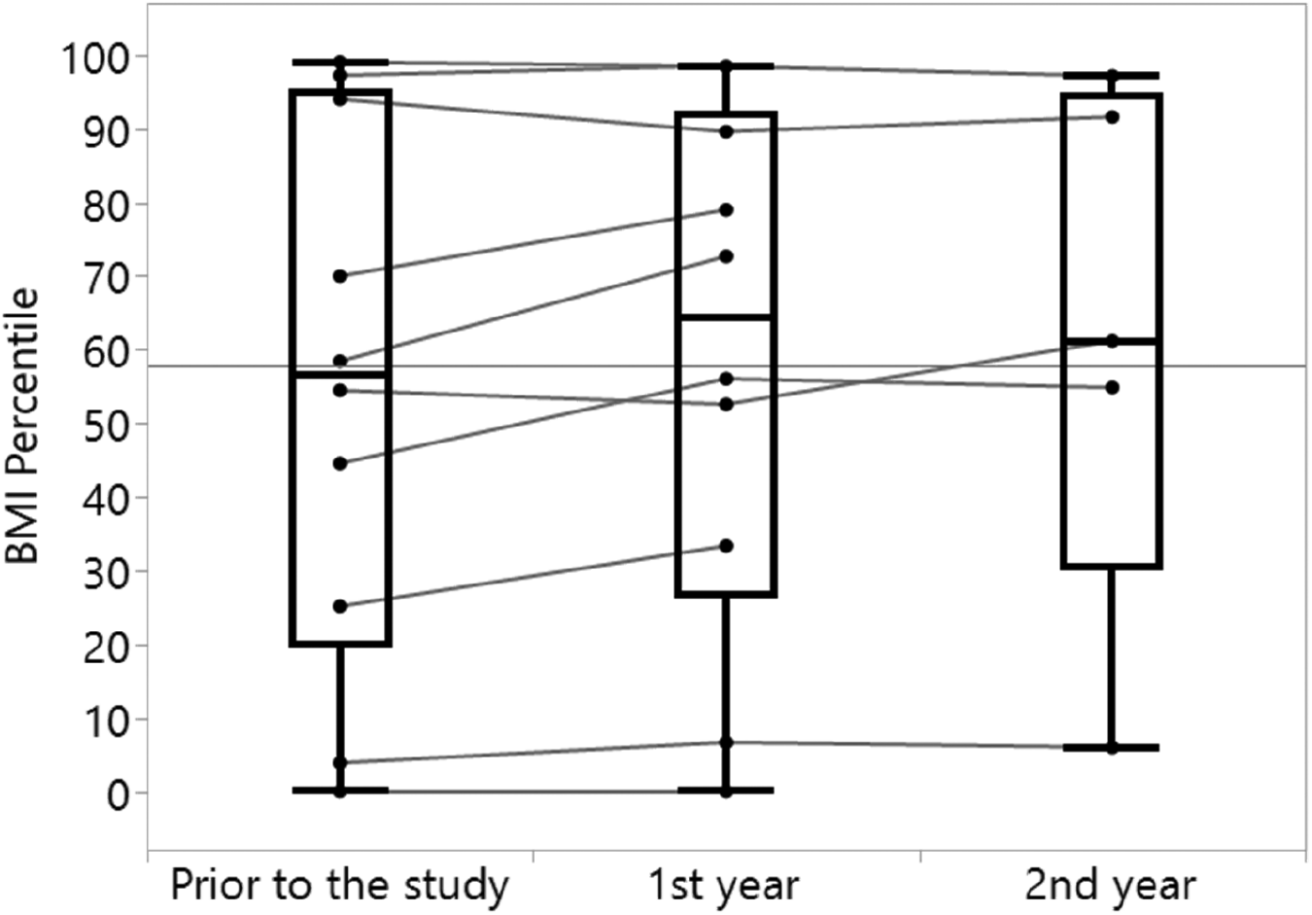
Changes in BMI percentiles over time (*n* = 10).

Increases in both FEV_1_% and BMI percentile were observed over time, with the most prominent change being the increase in FEV_1_% in 80% of the analyzed subjects from a year prior to the study to the first year of the study (Table 3, Figure 3). Similar increase in BMI percentile was observed among 60% of the participants (Figure 4). After adjustment for changes in CFTRm during the study, estimated average FEV_1_% increased from 74.4% to 80.8% (*p* = 0.004) over the course of the study. Similarly, estimated BMI percentile increased from 53.5 to 59.0 (*p* < 0.001) (Table 4). Results were adjusted for change in medication, which was not significant in any of the models with FEV_1_ increase by 3.2% (SE: 2.1%, *p* = 0.12), and BMI percentile decreased by 1.5 (SE: 1.4, *p* = 0.30) after the change.

**Table 3:** Changes in FEV_1_% and BMI percentiles over time (*n* = 10).

Parameter	Measure	A year prior to the study	First year	Second year	Prior to the study vs. first year *p*-value	Prior to the study vs. second year *p*-value
FEV_1_%	Median (Q1–Q3)	69.5 (47.5–98.2)	76.5 (58.5–103.5)	72 (58.5–106)	0.023	0.063
	Mean (SD)	72.9 (24.1)	79.1 (24.2)	80.2 (25.7)	—	—
BMI percentiles	Median (Q1–Q3)	56.4 (19.8–94.8)	64.8 (26.7–91.8)	61.2 (30.4–94.3)	0.256	0.437
	Mean (SD)	54.7 (36.6)	58.7 (35.8)	62.2 (36.3)	—	—

**Table 4:** Changes in FEV_1_ and BMI percentiles over time adjusted for change in CFTR modulators (*n* = 10).

Parameter^a^	A year prior to the study	First year	Second year	Prior to the study vs. first year *p*-value	Prior to the study vs. second year *p*-value
FEV_1_%	74.4 (7.7)	79.5 (7.7)	80.8 (7.7)	<0.001	0.004
BMI percentiles	53.5 (11.3)	58.2 (11.3)	59.0 (11.3)	<0.001	<0.001

^a^ Parameters are estimated means from the mixed-effect model with standard errors.

The platform enhanced communication between the patients and the caregivers: two patients were called into the clinic in real time and then hospitalized due to reports on ReX of abdominal pain and worse cough. Others were contacted by their CF physician when they did not adhere to their everyday regimen.

## Discussion

In this pilot study, we demonstrated that the use of the ReX platform by remote patient control in pwCFs treated by CFTRm is safe, easy to use, and effective in improving the adherence to this chronic and expensive treatment. To our knowledge, this is the first pilot report of the usage of the ReX platform in the medication management of a chronic disease.

Consensus on how to measure adherence is lacking, and the quality of studies addressing adherence in the CF population is generally poor. Overall, studies using self-reported measures yielded higher adherence scores than those that used objective measures (3). Current digital medication management methods of adherence assessment include reminder apps for mobile phone, embedded chip in packaging to detect pill removal, dispensing machines, and even a sensor incorporated in the pill itself to confirm it has been swallowed (14). There is also digital monitoring to inhaled therapies via chipped nebulizers plus tailored support via an online platform, which probably improves adherence and reduces treatment burden in pwCF (15). Using technological interventions for children with chronic lung disease suggest better adherence to treatment (16). However, such solutions focus on one aspect of the treatment cycle and have limitations. ReX is an innovative medication management platform designed to address the whole treatment cycle that involves several stages: packaging the medication in a safe mode; dispensing to the patient at the right time and in the right dose; and reminding patients to take their medication and monitoring impact of the medication on the patient's wellbeing. In this way, ReX assures the patient engagement with the process and consequently better adherence to the prescribed regimen. Improving adherence is a key factor in refining patient safety and quality of care tailored to patients’ needs, in reducing unused and improper medications, increasing the effectiveness and cost-effectiveness of healthcare, and consequently, improving the financial sustainability of health systems.

In the current study, we also found that by using the ReX platform, a statistically significant improvement in the clinical outcomes was achieved: FEV_1_% improved by 8.6% and BMI percentile by 10.2% after 2 years of treatment. Our cohort included pwCFs with different CFTR mutations and changes in CFTRm over time, and still found a significant improvement in FEV_1_%. This improvement is comparable to the absolute percentage change in FEV_1_ seen in phase 3 trials on pwCFs aged 12 years or older treated by ETI [14.3% by 6 months in pwCFs with a single Phe508del allele (17) or 10% by 4 weeks in pwCFs homozygous to Phe508del (18)] and on TI treatment [6.8% by 4 weeks in residual-function heterozygous (19) or 4% by 24 weeks in pwCFs homozygous to Phe508del (20)]. In these clinical studies, strict follow-ups and adherence diaries are maintained; therefore, adherence levels are high and resemble the adherence achieved by using the ReX platform.

There are some advantages in the current study: first is the double-step process of measuring adherence (record on cloud by the platform + pill accountability by team), which assures supreme precision; second is the long-term follow-up of a year to two, regarding adherence and clinical outcomes to CFTRm treatment by the novel ReX platform. However, our study has several limitations. First is the the heterogeneous and small group size. Second, a selection bias might have been inadvertently made by choosing subjects with relatively low–medium adherence and ending with all subjects being men. Another disadvantage was that the adherence to CFTRm in the year prior to the use of the ReX platform was based on patient self-reports and care team assessment. These are known to be unreliable methods. Also, adherence to other CF treatments including chest physiotherapy during the trial was made by patient and team assessments and not by the ReX platform.

In conclusion, we demonstrated that using the ReX platform to monitor the CFTRm treatment in pwCFs is safe, easy to use, and effective in improving adherence to this chronic and expensive therapy, and consequently, in improving some clinical outcomes. It seems that it may potentially reduce costs to healthcare providers and prevent drug waste. Further larger and long-term studies are required to examine the clinical benefits of the ReX platform for other therapies in other chronic diseases.

## Data Availability

The raw data supporting the conclusions of this article will be made available by the authors, without undue reservation.
