# Supplementary material for: Optimizing CFTR modulator therapy management for cystic fibrosis through the ReX platform
Source: Front Pediatr. 2023 Dec 19;11:1300968. doi: 10.3389/fped.2023.1300968 (PMC10766369; doi:10.3389/fped.2023.1300968)
Supplement: Supplementary file 1 [file Datasheet1.pdf]

SUPPLEMENTARY MATERIAL

dosentrx ™

Assessment of the Rex Platform in the management of CF patients treated with CFTRm in the home setting and self-report

Year-1

Safety

Yes	No	Have you encountered the following problems while using the device?	
☐	☐	Overdose (for example, 2 pills were extracted together)	1
☐	☐	A pill falling from the device during inhalation (the pill did not enter the mouth but fell out)	2
☐	☐	Changing shape of the pill (for a broken or deformed pill)	3
☐	☐	Any other unusual event related to taking the drug through the platform	4

Comments:____

____

Functioning

Yes	No		
☐	☐	Have you always been able to take the medicine using the platform?	1
☐	☐	Have you tried reporting side effects using the platform?	2
		If the answer to question 2 is “yes”:	
☐	☐	Did you get to the relevant screen easily?	3
☐	☐	Were you able to report every time you tried?	4
☐	☐	Was there a specific side effect you wanted to report that did not appear in the options on the monitor? If so, please specify in the comments below	5
☐	☐	Did you encounter a technical problem while using the platform? If yes, please specify on the comments below	6

Comments:____

____

Usefulness

Yes	No		
☐	☐	During the use of the platform, did you always take the pill in the right dose and at the time according to the treatment plan?	1
☐	☐	Did you receive personal reminders (phone call/ text message)?	2
☐	☐	Did the personal reminders improve your compliance with the drug treatment?	3
☐	☐	Did the platform help you prevent mistakes (mistakes such as taking it at the wrong time, missing a dose, taking it in a dangerous situation, etc.)	4

☐ ☐ Did the platform help you follow the treatment instructions and report side effects? 5

Comments:____

____

Usability

Please mark the box that best describes your opinion on a 5-point scale.

We would be happy to receive details for marking boxes 4 and 5.

1=Easy and friendly 5=Hard

1	2	3	4	5	Irrelevant		
☐	☐	☐	☐	☐	☐	The treatment instruction on the platform screen	1
☐	☐	☐	☐	☐	☐	Inhaling the medicine using the platform	2
☐	☐	☐	☐	☐	☐	Performing cartridge exchange	3
☐	☐	☐	☐	☐	☐	Transfer of treatment data through the application on the mobile phone	4
☐	☐	☐	☐	☐	☐	Device charging frequency	5
☐	☐	☐	☐	☐	☐	Report side effects using the device	6

Details if necessary: ____

____

____

General opinion

Please mark the box that best describes your opinion.

1=Disagree 5=Completely Agree

1	2	3	4	5	Irrelevant		
☐	☐	☐	☐	☐	☐	The platform allowed me to report unusual events easily and conveniently	1
☐	☐	☐	☐	☐	☐	The platform gave me a sense of security knowing that the clinical staff is aware of my condition when I report using the device	2
☐	☐	☐	☐	☐	☐	The reminder method helped me comply with the treatment instructions	3
☐	☐	☐	☐	☐	☐	In general, I feel that my treatment is more effective with the platform than without	4
☐	☐	☐	☐	☐	☐	I would like to continue using the platform	5
☐	☐	☐	☐	☐	☐	I would recommend others to use the platform	6

We thank you for your cooperation!

Assessment of the Rex Platform in the management of CF patients treated with CFTRm in the home setting and self-report

Year-2

Yes	No	Please answer the following questions:	
☐	☐	I encountered an overdose (for example, 2 pills were extracted together)	1
☐	☐	A pill fell from the device during inhalation (the pill did not enter the mouth but fell out)	2
☐	☐	I encountered a changed shape of a pill (for a broken or deformed pill)	3
☐	☐	I encountered any other unusual event that is related to taking the drug through the platform	4
☐	☐	I received personal reminders (phone call/ text message)	5

Comments:____

____

General opinion

Please mark the box that best describes your opinion.

1=Disagree 5=Completely Agree

1	2	3	4	5	Irrelevant		
☐	☐	☐	☐	☐	☐	The platform is easy to use	1
☐	☐	☐	☐	☐	☐	The platform allowed me to report unusual events easily and conveniently	2
☐	☐	☐	☐	☐	☐	The reminders help me not to forget to take medication on time	3
☐	☐	☐	☐	☐	☐	In general, I feel that my treatment is more effective with the platform than without	4
☐	☐	☐	☐	☐	☐	I would like to continue using the platform	5
☐	☐	☐	☐	☐	☐	I would recommend others to use the platform	6

What was the greatest benefit for you in using the platform?

____

____

What would you change in the device or the method of treatment and follow-up with the Rex platform?

____

____

We thank you for your cooperation!
